# Supplementary material for: Meta‐analysis of clinical metabolic profiling studies in cancer: challenges and opportunities
Source: EMBO Mol Med. 2016 Sep 6;8(10):1134–42. doi: 10.15252/emmm.201606798 (PMC5048364; doi:10.15252/emmm.201606798)
Supplement: Supplementary file 1 — Expanded View Figures PDF [file EMMM-8-1134-s001.pdf]

Expanded View Figures

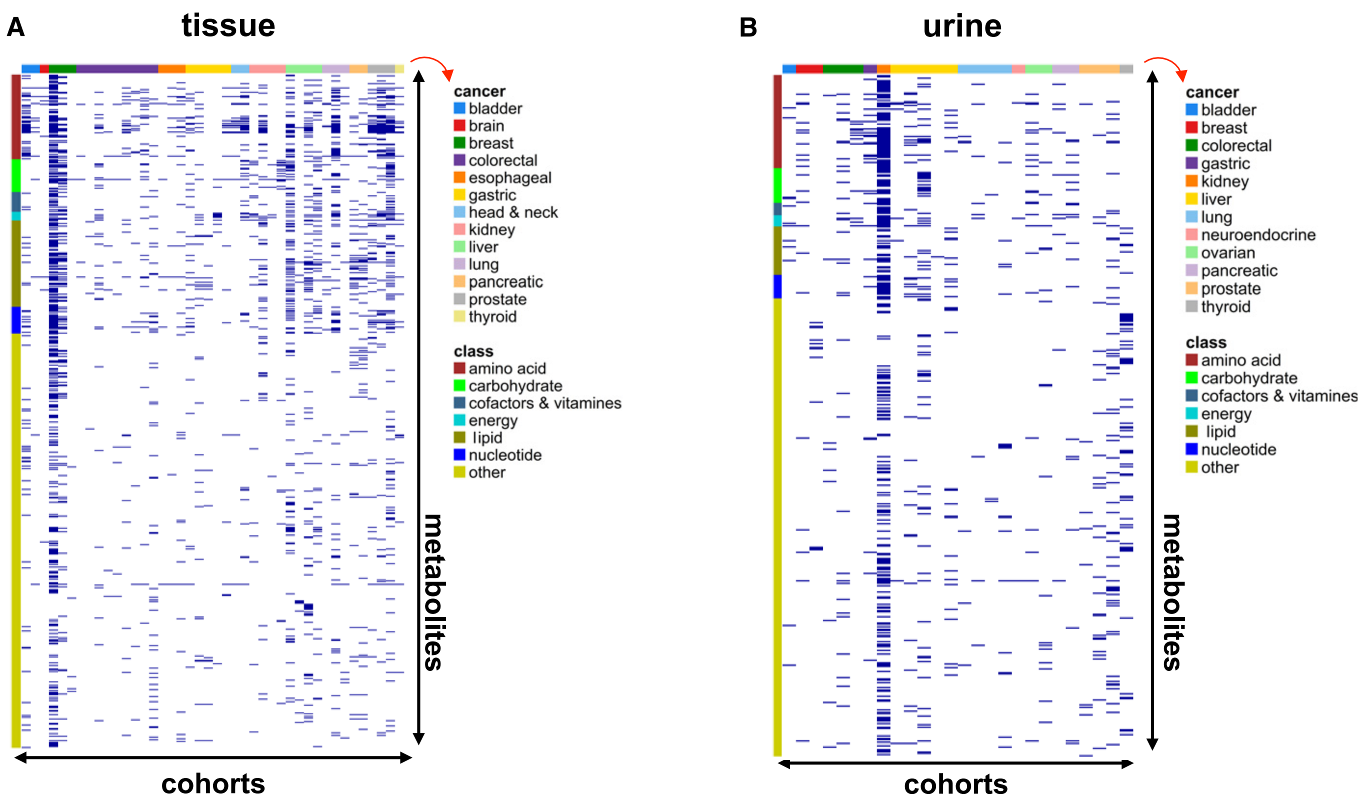

Figure EV1. Metabolite reporting in cancer tissue and urine.

A, B Heat maps of all metabolites measured in tissue (A) or urine (B) in various cancer types included in this study, illustrating that most individual studies report only a small subset of all previously measured metabolites (each dark blue mark denotes a metabolite that was reported to be increased or decreased). It is typically not described whether the metabolites that were not reported (white "empty" space) were not measured, or measured but not reported. Labeling: *x*-axis (top): ribbon color code, denoting the cancer type (right; indicated by red arrow); *x*-axis (bottom): cohorts, arranged from 1 to 39 (tissue, A) or from 1 to 26 (urine, B); *y*-axis (right): all 780 (tissue, A) or 549 (urine, B) metabolites reported in at least one of the studies; *y*-axis (left): ribbon color code, denoting the metabolite class. See also Tables EV3 and EV5.

 © 2016 The Authors

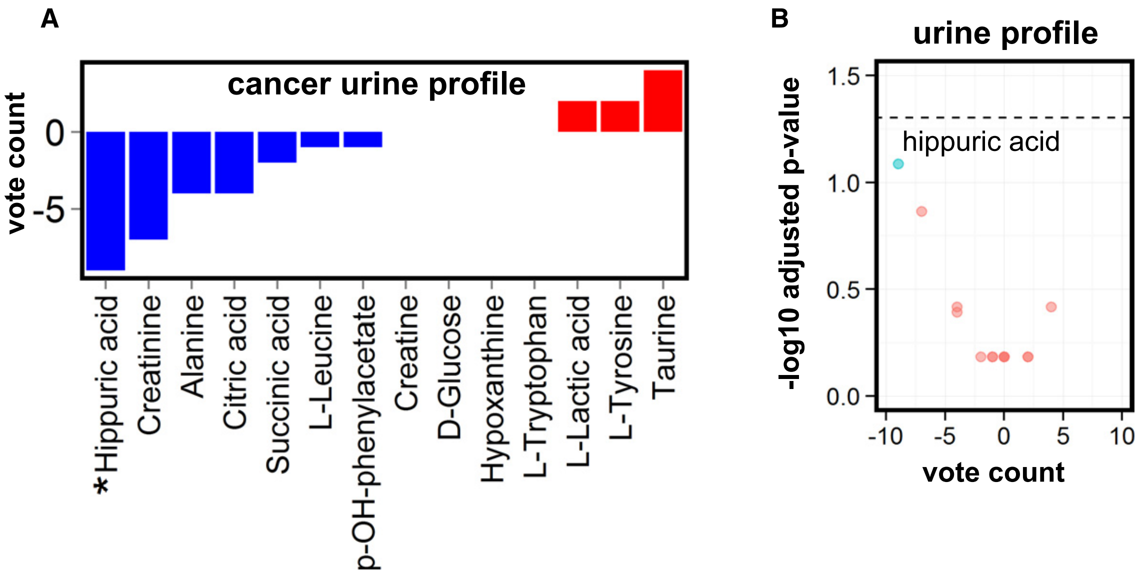

Figure EV2. Metabolic signatures of urine in cancer patients.

A Vote counting of urine metabolites, reported in at least six cohorts of cancer patients, reveals deregulated metabolites. Blue bars: decreased metabolites; red bars: increased metabolites. An asterisk (*) in front of the name of the metabolite indicates at least a statistical trend (P-value < 0.1) obtained using the sign test.

B Volcano plot of urine metabolites in cancer patients (reported in at least six cohorts) with the vote-counting score on the x-axis and the -log10 adjusted P-value on the y-axis. Cyan indicates deregulated metabolites that show a trend (P-value < 0.1) or statistical significance (P-value < 0.05; above black dashed horizontal line); red indicates metabolites with a P-value > 0.1 (see Table EV3 for full vote-counting tables, vote counting statistics were calculated using the sign test).

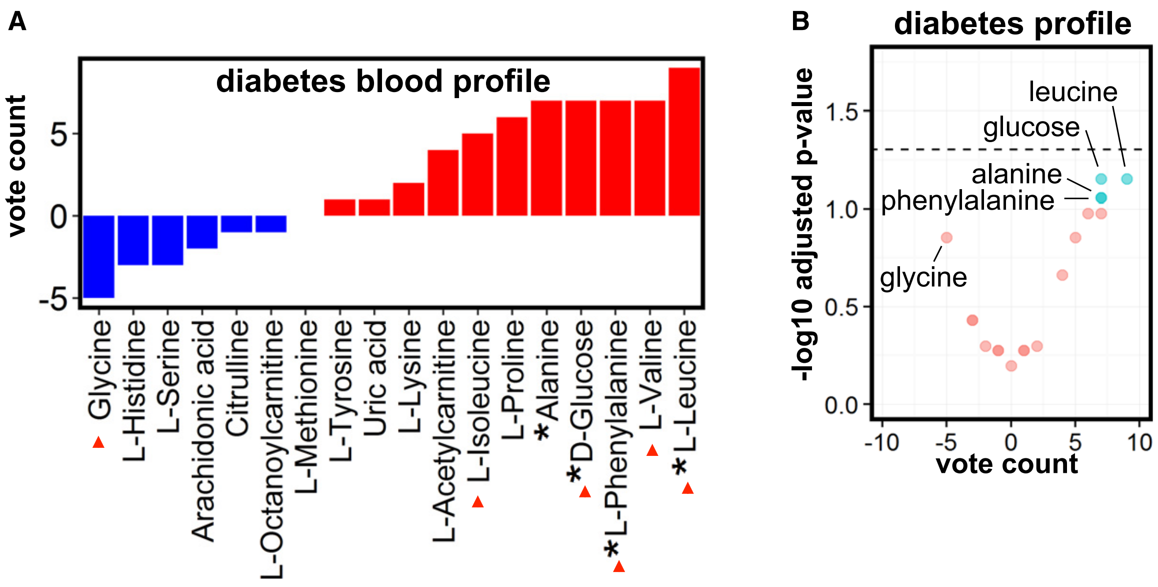

Figure EV3. Metabolic profiling in diabetes.

A Vote counting of blood metabolites in type 2 diabetes patients (reported in at least 6 cohorts) reveals deregulated metabolites. Blue bars: decreased metabolites; red bars: increased metabolites. An asterisk (*) in front of the name of the metabolite indicates a statistical trend (P < 0.1) obtained using the sign test.

B Volcano plot of blood metabolites in diabetes with the vote-counting score on the x-axis and the −log10 adjusted P-value on the y-axis. Cyan indicates deregulated metabolites that show a trend (P-value < 0.1) or statistical significance (P-value < 0.05; above black dashed horizontal line); red indicates metabolites with a P-value > 0.1 (see Table EV6 for full vote-counting tables, vote counting statistics were calculated using the sign test).

© 2016 The Authors
